# Genistein and Naringenin as Defense Molecules

**DOI:** 10.3390/molecules29235505

**Published:** 2024-11-21

**Authors:** Sylwia Goławska, Iwona Łukasik, Paweł Czerniewicz

**Affiliations:** Institute of Biological Sciences, Faculty of Natural Sciences, University of Siedlce, Prusa 14, 08-110 Siedlce, Poland; iwona.lukasik@uws.edu.pl (I.Ł.); pawel.czerniewicz@uws.edu.pl (P.C.)

**Keywords:** flavonoids, naringenin, genistein, black bean aphid, probing/feeding behavior

## Abstract

Genistein and naringenin, plant phenolic compounds, are recognized for their health benefits and role in plant defense against herbivores. However, little research exists on how these compounds affect aphid feeding, particularly that of the black bean aphid (*Aphis fabae* Scopoli) (Hemiptera: Aphididae), a major pest. This study aimed to evaluate the effects of genistein and naringenin, applied in vitro at different concentrations, on the feeding behavior of *A. fabae*. Statistical analysis indicated that both the type and concentration of flavonoids significantly influenced aphid stylet activity, salivation, and ingestion. Higher concentrations of both compounds hindered feeding behavior. A longer initial probe was observed on gels containing the studied flavonoids. Genistein at 0.1% completely inhibited salivation while at 0.01%, it reduced the duration of salivation activities. Both compounds also delayed the start and lengthened the duration of active ingestion, though *A. fabae* tolerated genistein better than naringenin. Naringenin’s effects on feeding behavior were more pronounced at higher concentrations. These findings suggest that genistein and naringenin could be valuable chemicals to protect plants from aphids in a sustainable and environmentally friendly way.

## 1. Introduction

Due to the problems associated with pesticide use when it comes to human health, animals, and the environment, researchers have increasingly focused on developing plant-based pesticides, known as biopesticides, for pest control, and bioinsecticides for managing field, storage, domestic, and structural insects [1]. The demand for alternative, more effective, and environmentally friendly control agents or insecticides is high [2]. The use of natural plant parts and products as biopesticides to overcome the hazards of synthetic chemicals is now considered the best control measure, gaining widespread popularity. This has led to the recognition of plant secondary compounds as excellent alternatives to synthetic pesticides [3,4].

Plants, being immobile, have evolved chemical defenses against herbivores. They synthesize a range of secondary compounds, such as phenolics, which are products of the shikimate, phenylpropanoid, and flavonoid pathways. These metabolites play roles in pigmentation, growth, reproduction, and resistance to pathogens and herbivores [5]. Their modes of action against insects include repellency, antifeedant activity, oviposition deterrence, the inhibition of growth and development, toxicity, and, in some cases, sterility and death [6,7]. Consequently, these compounds can be integrated into pest management programs for crop protection [8]. Over 30,000 plant metabolites with insecticidal activities have been identified [9,10]. Toxic effects on insect pests are produced by compounds such as terpenoids, steroids, phenols, flavonoids, tannins, alkaloids, and cyanogenic glycosides [11]. Primarily, these compounds affect the midgut epithelium in insects while secondarily impacting their gastric caeca and Malpighian tubules, ultimately leading to antifeedant properties and death [12].

Flavonoids, in particular, are highly useful in pest management strategies due to their insecticidal properties [13]. These compounds protect plants from herbivorous insects by influencing insect behavior, growth, and feeding while also increasing mortality [14,15,16,17]. Flavonoids, such as those extracted from *Lonicera maacki*, act as feeding deterrents, with larvicidal activity against *Spodoptera exigua* [18]. Genistein- and rutin-rich soybean cultivars have been shown to disrupt the metamorphosis of *Piezodorus guildinii* [19]. Rutin also inhibits the larval growth of *Helicoverpa zea* and reduces the feeding of *Trichoplusia ni* larvae [20,21,22].

Furthermore, the synthesis of flavonoids can be induced by herbivores. For instance, infestations by *Aphis glycines* Matsumura (Hemiptera: Aphididae) on soybeans trigger the accumulation of daidzein, formononetin, and genistein [23]. Similarly, *Spodoptera litura* (F.) (Lepidoptera: Noctuidae) induces the synthesis of daidzein and formononetin in soybean leaves [24] while *Acyrthosiphon pisum* (Harris) (Hemiptera: Aphididae) promotes the accumulation of genistein in alfalfa [25]. The infestation of groundnut (*Arachis hypogaea* L.) plants by *Aphis craccivora* Koch induces the synthesis of quercetin, catechin, genistein, and other flavonoids [26].

Aphids, and other Hemiptera, feed on plant sap by penetrating plant tissues to reach the phloem sieve elements [27]. During this process, aphids may encounter plant allelochemicals, including flavonoids, which can influence their probing and feeding behavior [28,29]. Flavonoids can therefore affect both phases of aphid probing, depending on their concentration and composition. Studies have shown that increased concentrations of quercetin and luteolin negatively impact *A. fabae* feeding behavior [30] while high concentrations of luteolin and genistein reduce the diet ingestion of *A. pisum* [31].

**Figure 1:**
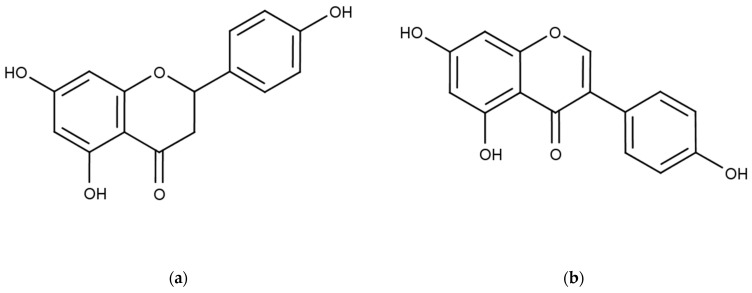
Chemical structures of naringenin (**a**) and genistein (**b**).

The present study was conducted in response to the need for biological control programs aimed at reducing environmental pollution. The goal was to evaluate the effects of two flavonoids, naringenin and genistein, on aphid stylet penetration activities using in vitro sucrose–agarose gel assays. Naringenin is 2, 3-dihydro-5, 7- dihydroxy-2-(4-hydroxyphenyl)–4H-1-benzopyran-4-one. Genistein is chemically known as 5,7-dihydroxy-3-(4-hydroxyphenyl) chromen-4-one (Figure 1). These flavonoids have been exploited for their beneficial effects on human health [32,33,34]. Genistein possesses several activities including anti-inflammatory [35,36], antioxidant [37], anticancer [38,39], and neuroprotective properties [40,41]. Naringenin exhibits antidiabetic [42], antiatherogenic, antidepressant [43], immunomodulatory [44], antitumor [45], anti-inflammatory, antioxidant [46], DNA-protective [42], hypolipidaemic [47] activities, as well as memory-improving effects.

However, the ecological roles of flavonoids extend beyond their health benefits, with flavonoids functioning as signaling and defense molecules in plants [48]. Genistein and naringenin, for instance, could play a crucial role in pest management strategies involving transgenic plants that express specific flavonoids [49]. Enhancing flavonoid synthesis or modifying their profiles in plants could make crops less attractive to herbivores, representing a promising biotechnological approach [26], and on the other hand, it will bring health benefits to human beings.

Flavonoids, known for their significant biological properties, are abundant in crop and medicinal plants, where their health benefits have led to efforts to boost their levels through traditional and biotechnological methods. Besides their therapeutic applications, flavonoids are believed to contribute to plant protection by acting as biopesticides. Although flavonoid activity has been studied, the mechanisms underlying their effects on insects remain incompletely understood. It is suggested that specific flavonoids may influence insect behavior differently, yet how increased flavonoid concentrations impact insect activity remains unclear. So, to inform the use of flavonoids, it is essential to clarify their effects on other organisms, such as aphids. This study extended our previous research on the influence of flavonoids on insect behavior, particularly feeding. We focused on the black bean aphid, a major agricultural pest and virus vector worldwide [50]. We hypothesized that flavonoid quantity and composition could alter the probing and feeding behavior of *A. fabae*. Using the Electrical Penetration Graph (EPG) technique to track aphid stylet activity in sucrose–agarose gel setups, we gathered continuous data on feeding, offering insights into how flavonoids impact aphid feeding behavior [51,52].

## 2. Results

**Figure 2:**
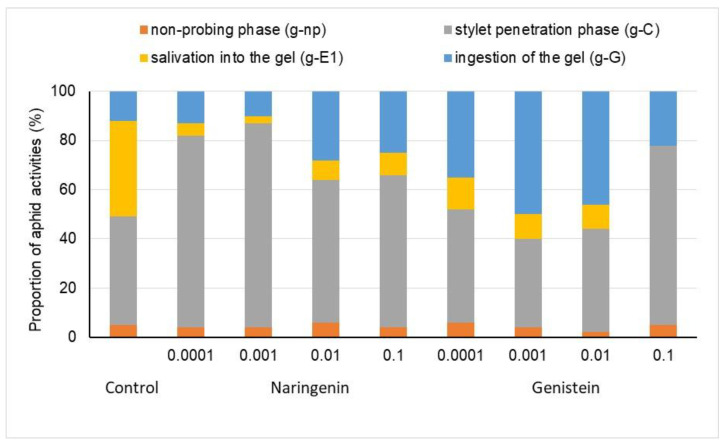
Proportions of time spent by aphids on probing/feeding activities on gels, as affected by naringenin and genistein during 2 h EPG monitoring.

EPG waveforms generated at the aphid–gel interface visualized major aphid activities, irrespective of compounds and their concentrations, including non-probing (aphid stylets outside the gel) activity, the stylet penetration phase (g-C), salivation into the gel (g-E1), and the ingestion of the gel (g-G) (Figure 2).

In *A. fabae*, the stylet probing activities on gels with naringenin and genistein occupied varied proportions of the 2 h experimental period. Generally, total probing times were greatest for naringenin at 0.001% and smallest for genistein at 0.001%. Aphids on naringenin at 0.001% spent over 83% of their time on stylet probing activities and approximately 4% on non-probing (Figure 2) activities. For the studied concentrations, the duration of salivation varied for both tested compounds. The addition of these chemicals to the gels resulted in either a reduction or complete inhibition of aphid salivation. On gels with genistein, salivation occurred only in *A. fabae* on gels with 0.0001%, 0.001%, and 0.01%, accounting for 13%, 10%, and 10% of EPG-recorded time, respectively (Figure 2). At 0.1%, genistein reduced the salivation time to zero. On gels with naringenin, salivation into the gel accounted for 3% to 9% of the recording time. The duration of ingestion also varied, with the highest values observed for genistein at 0.001% and 0.01% (50% and 46%, respectively) and for naringenin at 0.01% (28%) (Figure 2).

Concentrations of genistein significantly affected the time to the first g-C waveform (Kruskal–Wallis test; H_4,50_ = 30.27; *p* < 0.001) and duration of the first g-C waveform (Kruskal–Wallis test; H_4,50_ = 23.78; *p* < 0.001), but had no impact on the number of g-C waveforms (Kruskal–Wallis test; H_4,50_ = 2.45; *p* = 0.65). The timing to the first probe varied across genistein concentrations of 0.01%, 0.1%, and 0.0001% (multiple comparison test; *p* = 0.001 and *p* < 0.005). Differences were also observed for genistein concentrations of 0.001% compared to 0.01% and 0.1% (multiple comparison test; *p* = 0.011 and *p* < 0.005, respectively). Aphids initiated penetration most quickly on gels containing 0.1% genistein while the latest penetration was observed on gels with 0.0001% genistein (Table 1). Significant differences were found between the control and genistein concentrations of 0.01% and 0.1% (multiple comparison test; *p* = 0.005 and *p* < 0.005, respectively). Compared to the control, a longer initial probe was observed on gels containing genistein (Table 1). Similarly, naringenin concentrations significantly affected the time to the first g-C waveform (Kruskal–Wallis test; H_4,50_ = 16.35; *p* = 0.003) and the duration of the first g-C waveform (Kruskal–Wallis test; H_4,50_ = 14.96; *p* = 0.005). On gels containing 0.1% naringenin, aphids initiated penetration the fastest while on gels with 0.0001% naringenin, penetration occurred the latest (Table 1). The duration of the first probe was extended more than fourfold with 0.1% naringenin (multiple comparison test; *p* = 0.01) and more than twofold with 0.01% naringenin (multiple comparison test; *p* = 0.005) compared to the control (Table 1).

Genistein concentrations also significantly affected the time to the first g-E1 waveform (Kruskal–Wallis test; H_4,50_ = 26.86; *p* < 0.0001), the duration of the first g-E1 waveform (Kruskal–Wallis test; H_4,50_ = 26.92; *p* < 0.0001), and the average g-E1 time (Kruskal–Wallis test; H_4,50_ = 27.75; *p* < 0.0001). The addition of genistein to the gels resulted in reduced or completely inhibited aphid salivation (Table 1). At a concentration of 0.1%, genistein reduced the salivation time to zero (Table 1). Aphids feeding on gels with a higher concentration of genistein (0.01%) exhibited this waveform the earliest while it appeared the latest on gels with 0.0001% genistein (multiple comparison test; *p* < 0.001 in all cases). The duration of the first salivation tended to decrease with higher concentrations of genistein (multiple comparison test; *p* < 0.001). For aphids on gels with the lowest concentration (0.0001% genistein), the first salivation was observed after a longer time. A similar trend was noted for the average duration of g-E1, which generally decreased as the genistein concentration increased (multiple comparison test; *p* < 0.001) (Table 1). Naringenin concentrations similarly showed significant effects on the time to the first g-E1 waveform (Kruskal–Wallis test; H_4,50_ = 15.60; *p* = 0.0036), the duration of the first g-E1 waveform (Kruskal–Wallis test; H_4,50_ = 19.94; *p* = 0.0005), and the average g-E1 time (Kruskal–Wallis test; H_4,50_ = 17.58; *p* = 0.0015). Aphids feeding on gels containing 0.1% naringenin exhibited the g-E1 waveform the earliest while on gels with 0.01% naringenin, it appeared the latest (multiple comparison test; *p* < 0.05 in both cases). The duration of the first salivation was significantly shortened by 0.1% naringenin compared to the control (multiple comparison test; *p* < 0.05), and the average time of the g-E1 waveform on gels with naringenin was more than ten times shorter than in the control (Table 1).

Both genistein and naringenin concentrations significantly influenced the time to the first g-G waveform (Kruskal–Wallis test; genistein: H_4,50_ = 18.95; *p* = 0.0008; naringenin: H_4,50_ = 18.14; *p* = 0.0012), the duration of the first g-G waveform (Kruskal–Wallis test; genistein: H_4,50_ = 20.38; *p* = 0.0004; naringenin: H_4,50_ = 13.66; *p* = 0.0084), and the average g-G time (Kruskal–Wallis test; genistein: H_4,50_ = 23.55; *p* = 0.0001; naringenin: H_4,50_ = 16.87; *p* = 0.0021). Compared to the control group, aphids feeding on gels with genistein initiated their first active ingestion more than seven times later (multiple comparison test; *p* < 0.05). The duration of the first g-G waveform was extended sixfold with 0.01% genistein (multiple comparison test; *p* = 0.001) and fourfold with 0.001% genistein (multiple comparison test; *p* = 0.009). The average time of active ingestion also tended to increase with genistein, with statistically significant differences observed at concentrations of 0.001% (multiple comparison test; *p* = 0.005) and 0.01% (multiple comparison test; *p* < 0.005) (Table 1). Overall, the g-G waveform average duration on gels with genistein was more than six times longer than in the control for 0.01% genistein. Naringenin also delayed the appearance of the first g-G waveform. Significant differences were found between the control and naringenin concentrations of 0.001% and 0.01% (multiple comparison test; *p* < 0.005 and *p* = 0.022, respectively). Compared to the control, aphids on gels with 0.001% naringenin initiated their first active ingestion approximately seven times later, and five times later on gels with 0.01% naringenin (Table 1). Naringenin also tended to prolong active ingestion, with a more-than-twofold longer duration of the first active ingestion observed on gels with 0.01% naringenin (multiple comparison test; *p* = 0.014) (Table 1). On these gels, the average active ingestion time was approximately twice as long as in the control (multiple comparison test; *p* = 0.007). Statistical differences in the average g-G waveform time were observed for 0.001% and 0.1% naringenin (multiple comparison test; *p* = 0.014) (Table 1).

The statistical analysis showed that the effect depended on both the compounds and flavonoid concentrations (Table 2, Table 3 and Table 4). The tested compounds and their concentrations statistically affected stylet activity on sucrose–agarose gel (Table 2).

The analysis revealed significant effects of the tested compounds and concentrations on the total duration g-C waveform (GLM; F_7,72_ = 15.87; *p* < 0.001), the time to the first g-C waveform (GLM; F_7,72_ = 22.65; *p* < 0.001), and the duration of the first t g-C waveform (GLM; F_7,72_ = 5.39; *p* < 0.001). There was no effect of the compounds or concentrations on the number of g-C waveforms (GLM; F_7,72_ = 0.77; *p* = 0.613). The total time, time to the first, and duration of the first g-C waveform were significantly influenced by both the tested compounds and concentrations. Interactions between the tested factors were found for the total time of the g-C waveform and the time to the first g-C waveform (Table 2).

The tested compounds and concentrations also statistically affected *A. fabae* salivation (g-E1 waveform) on sucrose–agarose gel (Table 3). The analysis indicated significant effects of the tested compounds and concentrations on the total time of the g-E1 waveform (GLM; F_7,72_ = 3.67; *p* < 0.001), time to the first g-E1 waveform (GLM; F_7,72_ = 4.30; *p* < 0.001), and duration of the first g-E1 waveform (GLM; F_7,72_ = 10.46; *p* = 0<0.001), but no significant effect on the number of g-E1 waveforms (GLM; F_7,72_ = 1.916; *p* = 0.079). The analysis revealed an effect of the tested compounds on the duration of the first g-E1 waveform and an effect of the compound concentrations on both the time to the first g-E1 waveform and the duration of the first g-E1 waveform (Table 3). There was no effect of the concentrations on the total time of the g-E1 waveform and no effect of the compounds on the total time or time to the first g-E1 waveform (Table 3). Interactions between the tested factors were found for the total time of the g-E1 waveform, time to the first g-E1 waveform, and the duration of the first g-E1 waveform (Table 3).

The studied compounds and concentrations statistically affected *A. fabae* ingestion (g-G waveform) on the sucrose–agarose gel (Table 4).

The analysis showed significant effects of the tested compounds and concentrations on the number of g-G waveforms (GLM; F_7,72_ = 2.34; *p* = 0.033), total time of the g-G waveforms (GLM; F_7,72_ = 8.62; *p* < 0.001), time to the first g-G waveform (GLM; F_7,72_ = 2.46; *p* = 0.026), and the duration of the first g-G waveform (GLM; F_7,72_ = 6.09; *p* < 0.001). Both the compound and concentration were significant for the total time of the g-G waveform and the duration of the first g-G waveform. The compounds were significant for the time to the first g-G waveform (Table 4). There was no effect of the compounds or concentrations on the number of g-G waveforms. Interactions between the tested factors were found for the number of g-G waveforms, total time of the g-G waveform, and duration of the first g-G waveform (Table 4).

## 3. Discussion

Natural compounds derived from plants are particularly valuable in pest management strategies. Flavonoids, in particular, are especially effective due to their insecticidal properties. Additionally, secondary plant metabolites, such as diarylheptanoids and isocoumarins, both isomers of flavonoids, are recognized for their anti-herbivorous and insecticidal effects [53,54]. These compounds modulate the probing and feeding behaviors of insects, but detailed investigation on the mechanisms by which flavonoids modulate behavior, especially feeding behavior, remains unknown and their precise mode of insecticidal action is not fully understood [15,30,31,55]. In this study, we investigated the feeding response of *A. fabae* species to two flavonoids, naringenin and genistein, to explore the potential of these compounds as chemical barriers against aphids. Demonstrating the antifeeding effects of these compounds on aphids could lead to inhibiting aphid population growth, thus reducing damage caused by these pests. We aimed to determine whether the effectiveness depends more on the compound itself or on its concentration. Plant chemistry serves as a starting point for breeding pest-resistant cultivars [30,56]. In our research, we used fundatrigeniae, the most frequent morph of *A. fabae* infesting *V. opulus*, as this morph stays on the host plant for the longest period and plays a pivotal role in population development on primary hosts [57]. These morphs exploit their host extensively, leading to reductions in major nutritional compounds.

To monitor *A. fabae* activities during the probing of sucrose–agarose gels containing genistein and naringenin, we used the EPG (electropenetrography) method. Using the EPG technique, in combination with sucrose–agarose gels, facilitates understanding and addressing the challenges of using chemical deterrents against insects [31,55,58] as the agarose-sucrose gels mimic the tissues surrounding the sieve elements while a Parafilm M^®^ membrane containing sucrose syrup corresponds to sieve elements containing phloem sap [59]. This approach allowed us to focus on the effects of naringenin and genistein on aphid probing and feeding behavior. We analyzed parameters such as the total waveform duration (g-C, g-E1, and g-G waveforms), times to the first events, the durations of the first waveforms, and the numbers of probes. These parameters are reliable indicators of probing and feeding behaviors when aphids are exposed to chemical factors [60]. The use of the EPG to monitor the impact of chemical compounds on aphid probing and feeding behavior on sucrose–agarose gels has been previously demonstrated [30,31,52,55,58,61,62,63,64]. The waveforms generated in our study (g-np, g-C, g-E1, g-G) were consistent with previous findings. Similar to Goławska et al. [30,52], we did not observe a waveform analogous to passive ingestion on plants in our sucrose–agarose gels.

Our results indicated variations in aphid feeding behavior based on the flavonoid (naringenin or genistein) and its concentration (0.1%; 0.01%; 0.001%; 0.0001%). Differences were observed during different phases of probing and feeding, including the initial probe, stylet penetration into the sucrose–agarose gels, salivation, and ingestion.

The duration of probing, the number of probes, and the length of individual probes are indicators of factors that either stimulate or deter aphid probing during the pre-ingestive phase [65,66]. Aphids begin probing without delay in the absence of repellents [67]. During initial probes, aphids decide whether to continue or stop probing [28]. Our research demonstrated that both naringenin and genistein, along with their concentrations, influenced the time to first probing and its duration. While we observed an impact of these flavonoids on the total probing time, there was no significant interaction between the tested factors and the time of the first g-C waveform. Furthermore, no effect was found on the number of g-C waveforms. *A. fabae* exhibited reluctance to initiate feeding and the stylet penetration phase dominated during probing on gels containing genistein and naringenin. Probing activities accounted for 62% to 83% of *A. fabae* activity on gels with naringenin and 36% to 73% on gels with genistein. Deterrent factors present in the gels may have influenced this behavior [30,52,68].

The g-E1 waveform, indicating salivation into the gel, is analogous to watery saliva excretion on plants [51,52,69]. During this phase, aphids excrete saliva, containing various enzymes, which may aid in inactivating plant allelochemicals [70,71]. In our study, the g-E1 phase constituted up to 13% of total activity on gels with genistein, compared to a maximum of 9% on gels with naringenin at 0.1%, and as little as 3% at 0.001%. Notably, *A. fabae* displayed g-E1 activity on all gels with naringenin and genistein at concentrations ≥ 0.01%. At a 0.1% concentration, aphids did not excrete saliva into the gel, consistent with previous studies [31,55]. Higher concentrations of these compounds inhibited or significantly reduced aphid salivation. We found statistically significant effects of the compounds and their concentrations on the total g-E1 waveform duration, the time to the first g-E1 waveform, and the duration of the first g-E1 waveform.

Active ingestion (g-G waveform) from the sucrose–agarose gels was also influenced by naringenin, genistein, and their concentrations. Active ingestion from a gel is analogous to xylem sap ingestion in plants [52], where aphids ingest sap for osmoregulation, especially hydration [28,72]. Flavonoids appeared to prolong the ingestion time. The g-G waveform occupied a relatively large portion of the total probing time. On gels containing naringenin, g-G activity ranged from 10% to 28% while it was 35% to 50% on gels with genistein. This suggest that flavonoids at the tested concentrations stimulated ingestion, rather than inhibiting it. Our findings align with earlier reports [31,55], where genistein and naringenin prolonged g-G time for *A. pisum*. We observed significant effects of the tested flavonoids and their concentrations on the total g-G time, the time to the first g-G waveform, and the duration of the first g-G waveform. Similar delays in reaching the phloem phase were reported by Kordan et al. [73] in the peach potato aphid after treatment with naringenin derivatives. Conversely, Stec et al. [74] reported no effect of genistein on *A. pisum* probing behavior.

The detrimental effects of naringenin and genistein on aphid feeding behavior have significant consequences. The negative impact of flavonoids on herbivore performance has been well documented. Naringin has been shown to be toxic to the nymphs and apterous adults of the woolly apple aphid, *Eriosoma lanigerum* (Hausmann), leading to increased mortality [75]. Goławska et al. [55] found that naringenin decreased fecundity and increased mortality of *A. pisum* while it also reduced the growth rate of the late-season species *Nipaecoccus viridis* [76]. Del Rio et al. [77] identified naringenin as the most effective flavonoid against *Phytophthora citrophthora*. Additionally, naringenin inhibits the development of *A. craccivora* and other herbivores [78]. Genistein, on the other hand, has been shown to reduce the number of offspring produced by the *Formosan subterranean* termite [79] and to decrease insect longevity and overall fitness [22]. Genistein has been linked to reduced weight and activity in the clover root borer (*Hylastinus obscurus*), too [80].

As we can see, the feeding behavior of *A. fabae* is influenced by both naringenin and genistein and their concentrations. The observed deterrent effects of flavonoids on aphid feeding have broader implications, particularly in pest management. The higher the concentration is, the more pronounced the effect on aphid behavior will be. The application of genistein and naringenin can be considered against *A. fabae* because they shows potential to discourage the black bean aphid from probing and feeding. However, further experiments are necessary to determine the practical applicability of genistein and naringenin in integrated pest management strategies for aphids to understand the full range of activity of flavonoids both produced naturally and/or applied artificially.

## 4. Materials and Methods

### 4.1. Aphids

Fundatrigeniae, the most frequent morph of black bean aphids, *A. fabae* (Hemiptera: Aphididae), among those infesting the winter host *Viburnum opulus*, were used in this study. Aphid samples were collected randomly in the third decade of May 2022 from a wild population occurring on *V. opulus* located in green areas around Siedlce, Poland (52°12′ N, 22°17′ E). The climate of the area is characterized by an annual mean temperature of 8.7 °C, mean annual relative humidity of 79%, and total rainfall of 526 mm (source: https://en.tutiempo.net); (accessed on 10 October 24). During the experimental period, the weather was typical for spring in eastern Poland.

### 4.2. Chemicals and Gels

Genistein, isoflavonoid, was purchased from Fluka (Cat. No. 446720, Gillingham, UK) and naringenin, flavanone, from Sigma-Aldrich (Cat. No. 67604482, St. Louis, MO, USA). The effects of flavonoids on black bean aphid feeding behavior were investigated in vitro using sucrose–agarose gels. Gels were prepared by incorporating 1.25% agarose (Sigma A-0169, St. Louis, MO, USA) into a 30% sucrose solution and then adding one of the flavonoids to obtain final concentrations of 0% (control), 0.1%, 0.01%, 0.001%, and 0.0001%. After the mixtures were stirred, they were heated in a water bath at 75 °C for 30 min, then poured into plastic rings (10 mm high and 15 mm in diameter) covered with a stretched Parafilm M^®^ membrane (Arlington, TX, USA). The transparent gels formed after 1–2 min and were offered to aphids for probing.

### 4.3. Aphid Probing and Feeding Behavior

Electrical penetration graphs (EPGs) [81] were used to monitor the probing and feeding behavior of adult aphids exposed to flavonoids in the gel. The EPG technique allows the recording of different waveform patterns related to aphid activities and stylet locations during penetration, typically within plant tissues [82]. EPG waveforms were recorded in a Faraday cage under laboratory conditions adequate to those monitored in the green areas (approximately 21 ± 1 °C and 70% RH). Apterous adults were collected between 6 and 7 a.m. and dorsally tethered on the abdomen with a gold wire (2 cm long, 20 μm in diameter) and water-based conductive silver paint (Demetron, L2027, Darmstadt, Germany). After the insects were starved for 2 h to recover from tethering, EPGs were initiated (between 9 and 10 a.m.) by carefully transferring the aphids to sucrose–agarose gels and placing them individually in the centers of the membranes (one aphid per gel). A second electrode (a copper wire, 9 cm long, 1 mm in diameter) was inserted into each gel. Aphids were connected to a Giga-4 EPG amplifier (Wageningen, Agricultural University, Entomology Department, The Netherlands) coupled to an IBM-compatible computer through a DAS 8 SCSI acquisition card (Keithley, Cleveland, OH, USA). EPG recordings were conducted under continuous laboratory lighting, with recordings made for 10 aphids on 10 separate gels. Aphid probing and feeding behavior were monitored for 2 h.

EPG data were acquired and analyzed with STYLET 2.2 software (W.F. Tjallingii, Wageningen, The Netherlands). Waveforms were identified according to those found in sucrose–agarose gels [31,52,55,62] by analogy to those defined and described for plants [29,69,81]. The following patterns were distinguished: g-np (non-penetration), where the aphid’s stylet was outside the gel (analogous to the stylet being outside plant tissues); g-C (stylet pathway phase), representing stylet activity in the gel (analogous to intercellular movement through mesophyll or vascular tissues in plants and the formation of the salivary sheath); g-E1 (sieve element phase), indicating salivation into the gel (analogous to salivation into phloem sieve tubes in plants); and g-G (xylem phase), indicating ingestion from the gel (analogous to active ingestion from xylem). EPG parameters (number, total time, time to the first waveform, and time of the first waveform) on the studied activities (stylet activity, salivation, ingestion) were measured on gels with different concentrations of naringenin and genistein and used for statistical analyses.

### 4.4. Statistical Analysis

The EPG parameters, including the duration of stylet activity in the gel, the duration of salivation into the gel, and the duration of active ingestion from the gel, were analyzed using the Kruskal–Wallis test. To determine the effects of flavonoids on waveforms, we used a general linear model (GLM) with a Gaussian distribution. In the analysis response, variables were the individual waveforms and compounds, concentrations, and interactions between these variables were predictor variables. All statistical analyzes were performed using Statistica 12.0 (StatSoft, Krakow, Poland).

## 5. Conclusions

Naringenin and genistein are naturally occurring flavonoids known to possess various biological activities. The role of flavonoids in the prevention and treatment of diseases has been well established. However, their activity is not limited to health benefits; they also play a key role in a wide range of ecological interactions in plants, such as serving as defense molecules. Our research shows that the feeding behavior of *A. fabae* is influenced by both naringenin and genistein, with the effect becoming more pronounced as their concentrations increase. These compounds, as distinct chemical agents, have the potential to be used as insecticides and could be integrated into aphid control programs. Flavonoid-based biopesticides offer promising potential for sustainable agriculture. They are environmentally friendly and could reduce dependency on synthetic pesticides. Despite initial high production costs, flavonoid-based biopesticides may become more viable with advancing new technologies and environmental demand, offering benefits such as biodegradability, lower toxicity to non-target organisms, and slower resistance development in pests. Overall, flavonoid-based biopesticides demonstrate high potential as sustainable alternatives, especially in organic farming.

Thus, our findings offer valuable insights for plant breeders and plant protection services aiming to protect crops from aphids in a sustainable and environmentally friendly manner.

Furthermore, with advancements in genetic modification, it is now possible to produce flavonoids on a large scale, and their industrial applications, as you can see, extend far beyond nutraceuticals and pharmaceutical candidates. It is important to note, however, that studies on both the health effects and the bioavailability of flavonoids at varying doses are still needed. Any bioactive compound may have adverse effects or different outcomes depending on the dosage. This should be taken into consideration when genetically modifying plants to enhance the flavonoid content and their use in biological preparations.

## Figures and Tables

**Table 1:** *Aphis fabae* activity (g-C, g-E1, g-G waveforms) on an sucrose–agarose gel as affected by genistein and naringenin.

Flavonoid Added	Concentration (%)	Number of Waveforms/2 h	Time to the First Waveform (min)	Duration of the First Waveform (min)	Average Time of Waveform (min)
			g-C waveform		
Control	0.00	4.70 ± 1.86	3.66 ± 1.54	7.25 ± 5.80	19.26 ± 5.76
Genistein	0.0001	4.70 ± 0.96	4.78 ± 0.52 a	12.89 ± 2.79	17.50 ± 4.03
	0.001	4.00 ± 0.42	3.85 ± 0.14 a	13.11 ± 2.49	12.21 ± 1.94
	0.01	4.00 ± 0.88	0.79 ± 0.14 b	28.35 ± 6.82 *	19.22 ± 5.55
	0.1	3.30 ± 0.45	0.53 ± 0.09 b	45.15 ± 5.86 *	31.28 ± 4.63
Naringenin	0.0001	4.50 ± 0.26	4.34 ± 0.26 AB	9.76 ± 1.25	21.90 ± 1.96
	0.001	5.30 ± 0.28	4.15 ± 0.24 AB	12.01 ± 4.59	19.68 ± 1.67
	0.01	5.60 ± 1.19	4.79 ± 0.57 A	17.94 ± 3.69 *	16.84 ± 2.99
	0.1	5.30 ± 1.49	1.47 ± 0.55 B	29.14 ± 8.31 *	32.28 ± 7.65
			g-E1 waveform		
Control	0.00	3.40 ± 1.38	16.77 ± 6.98	18.02 ± 6.68	25.20 ± 7.04
Genistein	0.0001	2.20 ± 0.60	30.91 ± 3.76 a	12.89 ± 2.80 a	10.89 ± 2.40 a
	0.001	2.40 ± 0.55	25.05 ± 6.59 a	5.80 ± 0.74 a	5.82 ± 0.73 a
	0.01	2.60 ± 0.79	17.82 ± 7.05 a	3.68 ± 1.40 a	3.46 ± 1.04 a
	0.1	0.00 ± 0.00	0.00 ± 0.00 b	0.00 ± 0.00 b	0.00 ± 0.00 b
Naringenin	0.0001	1.80 ± 0.13	15.19 ± 1.81 AB	3.52 ± 0.32 A	3.25 ± 0.24 AB
	0.001	2.50 ± 0.32	16.21 ± 4.79 AB	2.05 ± 0.34 AB	1.98 ± 0.24 *AB
	0.01	3.00 ± 0.99	33.09 ± 8.11 A	4.50 ± 0.51 A	4.36 ± 0.53 A
	0.1	3.70 ± 1.41	7.04 ± 4.04 B	0.96 ± 0.40 *B	1.54 ± 0.55 *B
			g-G waveform		
Control	0.00	0.80 ± 0.40	5.89 ± 5.46	7.71 ± 5.92	8.54 ± 5.48
Genistein	0.0001	1.60 ± 0.25	45.89 ± 8.86 *	31.84 ± 7.16	31.79 ± 7.18 ab
	0.001	1.80 ± 0.13	44.09 ± 5.58 *	32.64 ± 4.70 *	36.10 ± 4.32 *ab
	0.01	1.10 ± 0.09	63.62 ± 8.30 *	49.29 ± 9.32 *	51.34 ± 8.73 *a
	0.1	2.00 ± 0.49	45.03 ± 9.50 *	13.41 ± 3.62	12.93 ± 3.44 b
Naringenin	0.0001	1.70 ± 0.15	24.65 ± 4.00	12.03 ± 1.55 *	9.38 ± 1.20 AB
	0.001	2.10 ± 0.17	42.64 ± 6.73 *	6.67 ± 1.01	5.26 ± 0.59 B
	0.01	2.20 ± 0.28	32.59 ± 5.97 *	15.28 ± 2.92 *	16.88 ± 2.57 *AB
	0.1	1.10 ± 0.29	28.99 ± 8.50	20.33 ± 7.27	23.28 ± 6.89 A

Values were derived from 2 h EPG recordings and are means ± SE; *n* = 10. Values marked with an asterisk differed from the controls at *p* < 0.05 (Kruskal–Wallis test). For each given model, different letters in columns denote significant differences between the concentrations of compounds (naringenin and genistein) (Kruskal–Wallis test, *p* < 0.05).

**Table 2:** Statistical results of the GLM of *A. fabae* stylet activity (g-C waveform) on sucrose–agarose gel as affected by genistein and naringenin (analysis based on 80 cases grouped into 8 categories).

Waveform g-C	F_7,72_	*p*-Value
Number		
Compound	3.39	0.069
Concentration	0.11	0.955
Compound × concentration	0.57	0.639
Total time		
Compound	43.89	<0.001
Concentration	5.68	<0.001
Compound × concentration	16.72	<0.001
Time to the first		
Compound	19.51	<0.001
Concentration	33.57	<0.001
Compound × concentration	12.78	<0.001
Duration of the first		
Compound	4.32	<0.05
Concentration	10.48	<0.001
Compound × concentration	0.85	0.473

**Table 3:** Statistical results of the GLM of *A. fabae* salivation (g-E1 waveform) on sucrose–agarose gel as affected by genistein and naringenin (analysis based on 80 cases grouped into 8 categories).

Waveform g-E1	F_7,72_	*p*-Value
Number		
Compound	2.98	0.089
Concentration	0.62	0.605
Compound × Concentration	2.89	0.053
Total time		
Compound	1.08	0.301
Concentration	1.86	0.144
Compound × Concentration	6.35	<0.001
Time to the first		
Compound	0.03	0.885
Concentration	6.64	<0.001
Compound × Concentration	3.38	<0.05
Duration of the first		
Compound	10.56	<0.001
Concentration	13.10	<0.001
Compound × Concentration	7.79	<0.001

**Table 4:** Statistical results of the GLM of *A. fabae* active ingestion (g-G waveform) on sucrose–agarose gel as affected by genistein and naringenin (analysis based on 80 cases grouped into 8 categories).

Waveform g-G	F_7,72_	*p*-Value
Number		
Compound	0.59	0.443
Concentration	0.79	0.501
Compound × concentration	4.48	<0.05
Total time		
Compound	29.81	<0.001
Concentration	3.41	<0.05
Compound × concentration	6.77	<0.001
Time to the first		
Compound	10.01	<0.05
Concentration	1.15	0.335
Compound × concentration	1.25	0.297
Duration of the first		
Compound	20.11	<0.001
Concentration	2.74	<0.05
Compound × concentration	4.77	<0.05

## Data Availability

Data are contained within the article.
